# Effects of SGLT2 inhibitors on gout-related and cardiometabolic outcomes in patients with type 2 diabetes mellitus: a scoping review

**DOI:** 10.1186/s42358-026-00564-w

**Published:** 2026-07-24

**Authors:** Henrique Pereira Sampaio, Barbara Casarin Henrique-Sanches, Carlos Antonio Negrato

**Affiliations:** Bauru School of Medicine, University of São Paulo, Bauru, Brazil

**Keywords:** Gout, Diabetes mellitus, type 2, Sodium-glucose transporter 2 inhibitors, hyperuricemia, Cardiovascular diseases, Mortality, Scoping review

## Abstract

**Background:**

This review aimed to map the effects of sodium-glucose cotransporter 2 inhibitors (SGLT2i) on gout-related and cardiometabolic outcomes in patients with type 2 diabetes mellitus (T2DM) and gout.

**Methods:**

A scoping review was conducted following the Joanna Briggs Institute methodology and the PRISMA-ScR checklist. Searches were performed in PubMed, EMBASE, Scopus, Web of Science, LILACS/BVS, and gray literature sources. Eligible studies included patients with T2DM and gout treated with SGLT2i, assessing outcomes such as mortality, gout flares, serum uric acid (SUA) levels, emergency care visits, and hospitalizations due to gout. The PRISMA-ScR checklist is provided in Additional file 1, and the full search strategy in Additional file 2.

**Results:**

Of 282 articles initially identified, 22 met the inclusion criteria. Most studies were retrospective cohort studies (*n* = 12) or post hoc analyses (*n* = 8). Only three studies evaluated patients with both T2DM and gout exclusively. The main findings included reductions in SUA levels (*n* = 8), incidence of gout (*n* = 12), gout flares (*n* = 8), initiation of urate-lowering therapy or colchicine (*n* = 8), and gout-related emergency care or hospital visits (*n* = 1). Reductions in all-cause (*n* = 5) and cardiovascular (CV) mortality (*n* = 4) were also reported. Across the included studies, SGLT2i were frequently associated with favorable effects on the analyzed outcomes.

**Conclusion:**

SGLT2i appears to be associated with multiple favorable effects in patients with T2DM and gout, including urate-lowering effects, reduced gout flares, and lower mortality (all-cause and CV-related). Although limited by the small number of studies focusing specifically on this population, these findings support the potential therapeutic relevance of SGLT2i in patients with coexisting T2DM and gout, contributing to the management of both conditions and the reduction of CV-related events associated with them.

**Supplementary Information:**

The online version contains supplementary material available at https://doi.org/10.1186/s42358-026-00564-w.

## Background

Diabetes mellitus (DM) represents a serious global public health challenge, causing significant negative impacts on patients, health systems, and the global economy. According to the International Diabetes Federation [1], in 2024, approximately 589 million people between the ages of 20 and 79 lived with the disease, with projections indicating an increase to 853 million by 2050, especially in low- and middle-income countries. Approximately 90% of DM cases are type 2 diabetes mellitus (T2DM), a complex condition resulting from the interaction between genetic, environmental, socioeconomic, and lifestyle-related factors [2].

Gout is the most common inflammatory arthropathy in adults and is caused by chronic deposition of monosodium urate crystals in tissues and joints. Its clinical manifestations result from the host’s inflammatory response to this deposition [3]. Hyperuricemia is the main risk factor for gout [4], defined by the presence of serum uric acid (SUA) levels above 6.8 mg/dL (>0.41 mmol/L), which is the approximate limit of urate solubility [5]. This is a necessary but not sufficient condition for developing gout, as most individuals with hyperuricemia do not develop clinically evident disease [6, 7]. These individuals are classified as having asymptomatic hyperuricemia.

Several conditions that cause hyperuricemia are also considered risk factors for gout. These include metabolic syndrome, the use of hyperuricemic medications (such as diuretics and cyclosporine), and chronic kidney disease. Dietary habits also play a role, especially the consumption of purine-rich animal-derived foods and fructose-sweetened beverages. Alcohol intake and genetic predispositions are additional contributing factors [4].

The prevalence of gout varies widely, being approximately 4% in the United States and Canada, 1–4.75% in Europe, and as high as 10.4% in specific subpopulations such as Taiwanese aborigines [8]. In Brazil, data on gout prevalence are lacking, although hyperuricemia affects approximately 31.9% of men and 4.8% of women [9]. In recent decades, an increase in gout prevalence has been reported, possibly related to unhealthy dietary habits, increased longevity, the use of hyperuricemic medications, chronic kidney disease, and metabolic syndrome [8].

Studies based on population databases indicate a higher prevalence of comorbidities such as hypertension, T2DM, obesity, chronic kidney disease (CKD) [10], and metabolic syndrome [11] in patients with gout. Additionally, the incidence of cardiovascular (CV) outcomes, including heart attack (HA), stroke, heart failure (HF) [10, 12], and venous thromboembolism [13], is greater. Importantly, gout, like T2DM, is an independent CV risk factor, associated with increased mortality from both CV [14–17] and non-CV causes [16, 17].

There is evidence suggesting that acute gout flares are associated with a transiently increased risk of adverse CV events, including fatal HA or stroke, particularly within the first 60 days following an episode [18, 19]

Sodium-glucose cotransporter 2 inhibitors (SGLT2i) are oral hypoglycemic agents that inhibit SGLT2 in the S1 segment of the proximal convoluted tubule of the nephron, leading to glycosuria and consequently lowering blood glucose levels [20]. In addition to glycemic control, SGLT2i exert pleiotropic effects, including blood pressure reduction, weight loss, improvements in cardiometabolic markers, favorable CV outcomes, renoprotection [20, 21] and reductions in SUA levels [21, 22].

Owing to their potential to lower SUA, data suggest a reduced risk of developing gout in T2DM patients treated with SGLT2i [23–25]. However, most studies did not report whether hyperuricemia was present before SGLT2i use, nor did they differentiate between asymptomatic hyperuricemia and gout.

Given the frequent coexistence of gout and T2DM, as well as their shared association with metabolic syndrome and their role as independent CV risk factors, it is important to consider strategic interventions for individuals affected by both conditions. The objective is not only to manage the diseases but also to reduce CV-related events.

While systematic reviews are designed to answer specific, narrow questions often involving homogeneous data for meta-analysis, the current evidence regarding SGLT2i in the context of T2DM and gout remains notably heterogeneous. Available studies vary significantly in terms of patient populations, comparison groups, outcomes, and methodological designs. Therefore, a scoping review was chosen as the most appropriate methodology to provide a broad mapping of the existing literature, identify key concepts, and clarify the current state of evidence without the constraints of a formal quantitative synthesis, which would be precluded by the lack of uniformity in available data.

Therefore, this scoping review aimed to map the available evidence and identify knowledge gaps regarding the use of SGLT2i in patients with T2DM and gout, particularly concerning overall and CV mortality, incidence of gout, frequency of gout flares, reduction in SUA levels, and rates of emergency room visits and gout-related hospitalizations.

## Methods

This scoping review was conducted following the standards of the Joanna Briggs Institute to map the literature on a specific topic [26]. To ensure methodological rigor, we followed the Preferred Reporting Items for Systematic Reviews and Meta-Analyses extension for Scoping Reviews (PRISMA-ScR), which includes a checklist of 20 essential and 2 optional items [27]. The completed PRISMA-ScR checklist is provided as Additional file 1.

This review was developed through the following sequential steps: definition and alignment of the objectives and formulation of the guiding question; development and alignment of the inclusion criteria with the objectives and the guiding question; description of the planned approach to evidence searching, selection, data extraction, and presentation; systematic search for evidence; selection of evidence in two stages (first by screening titles and abstracts, then by full-text reading); data extraction; data analysis; presentation of results; and summary of the evidence about the review objectives, drawing conclusions and noting the implications of the findings.

The guiding question was developed via the population, concept, and context (PCC) strategy, where the population was defined as patients with T2DM and gout, the concept referred to mortality rates and other clinical and laboratory outcomes, and the context was the use of SGLT2i. Therefore, the research question was as follows: “What are the clinical and laboratory outcomes in patients with T2DM and gout treated with SGLT2i?”

The search strategy was developed in collaboration with a professional librarian to ensure a comprehensive and accurate identification of relevant studies. To ensure transparency and reproducibility, the complete search strings for all databases are provided in Additional file 2, allowing the search to be fully replicated.

The search strategy was applied to the following databases: MEDLINE/PubMed, EMBASE, Scopus, Web of Science, and LILACS/BVS. Additionally, gray literature was explored by consulting thesis databases such as the Biblioteca Digital Brasileira de Teses e Dissertações (BDTD) and ProQuest. The search strategy combined controlled descriptors and/or keywords related to the topic, with no restrictions regarding publication date, and was limited to articles written in English, Portuguese, or Spanish. While this may introduce potential linguistic bias, these languages represented most of the global scientific output on SGLT2i and gout. Health descriptors (DeCS/MeSH), keywords, and their entry terms were used, and Boolean operators (AND, OR, NOT) were applied to optimize the search.

An initial search using the three PCC components yielded 42 studies. Owing to the limited number of results, we opted for a broader search using only the population (T2DM and gout) and the context (SGLT2i), which identified 282 articles. Each article was assessed to determine whether it met the inclusion criteria. The final search was completed on February 7, 2025. The search strategies and the number of articles retrieved from each database are detailed in Additional file 2.

All the articles were exported to the Rayyan platform in RIS format, except those from PubMed, which were exported in text format. The duplicate removal tool in Rayyan was used, resulting in 129 unique articles for screening. A manual search of the reference lists of initially selected studies was also performed, yielding three additional articles, for a total of 132 articles to be screened.

Eligible studies included original research articles and post hoc analyses that addressed the three PCC elements, answered the research question, and were published in English, Portuguese, or Spanish, without time restrictions. We excluded articles in other languages, those not addressing the research question, reviews, editorials, letters, pamphlets, oral presentations, abstracts, conference posters, and studies without full-text availability.

Two independent reviewers (HPS, BCHS), blinded to each other’s assessments, screened the titles and abstracts of the 129 articles (phase 1) and then conducted full-text screening (phase 2) to determine eligibility. Inter-reviewer agreement was assessed, and any discrepancies were resolved through discussion and consensus until 100% agreement was reached. When necessary, a third reviewer (CAN) was consulted to provide a final decision. Data extraction followed the same independent and blinded approach.

**Fig. 1:**
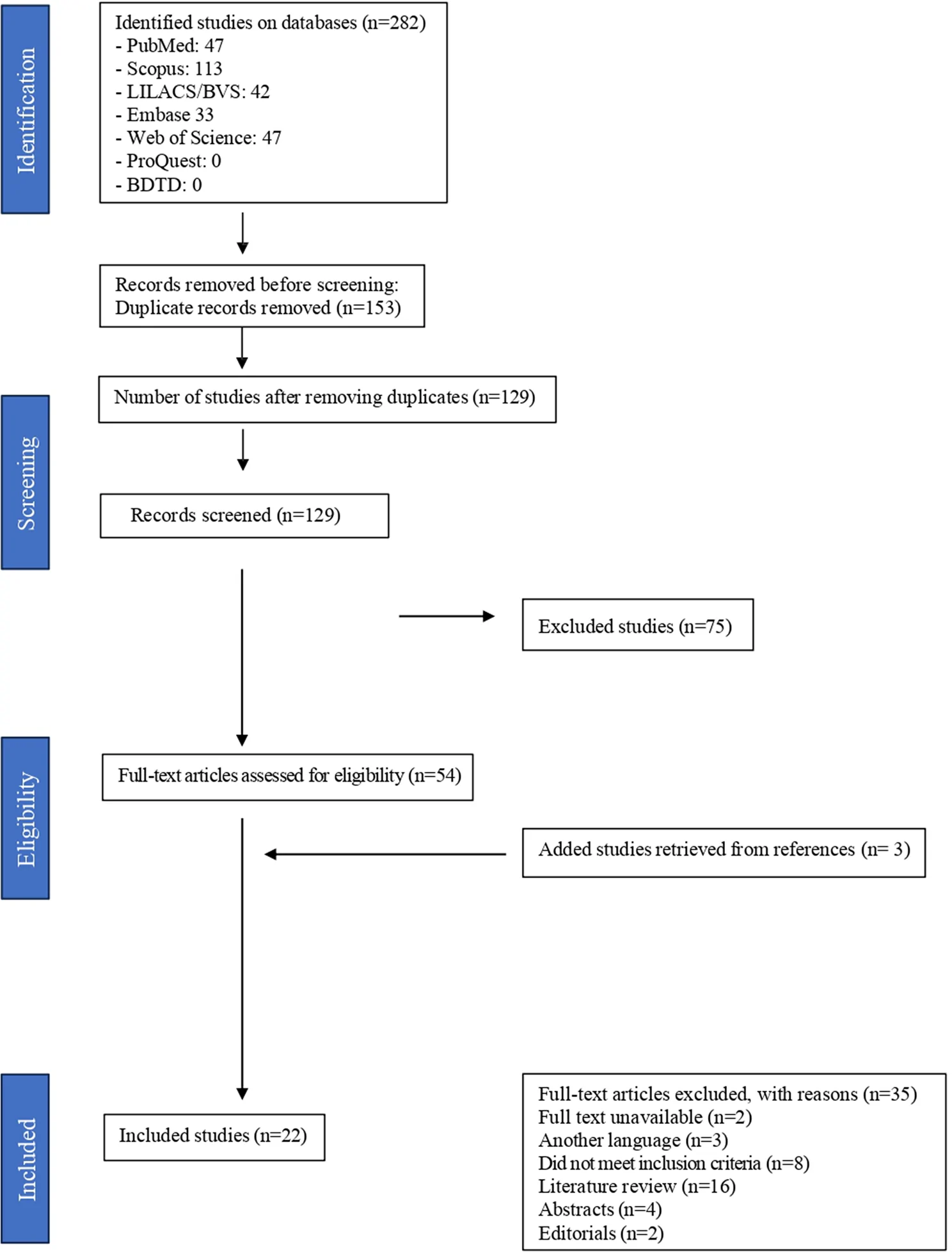
Flowchart of the article’s identification and selection process. Bauru 2025

In total, 22 articles met the predefined inclusion criteria and were included in this review. The selection process is summarized in the flowchart presented in Fig. 1. The included studies were numbered from 1 to 22.

Data were extracted using a standardized form developed by the authors in Microsoft Excel (2019 MSO - Version 2507 Build 16.0.19029.20136 - 32-bit), capturing the following information: author, year of publication, country of origin, journal, study design, population and sample size, percentage of individuals with gout at baseline, type of SGLT2i used, comparator (placebo or other antidiabetic drugs), outcomes analyzed, population characteristics (age, sex, ethnicity), CV comorbidities, mean follow-up time, and main findings of each study.

The results were analyzed descriptively and presented in narrative form and through summary tables, ensuring clarity and objectivity in data presentation. No formal critical appraisal of the individual studies was conducted, as the primary objective was to map existing evidence on the effects of SGLT2i in patients with T2DM and gout. Therefore, the focus was on descriptive analysis rather than methodological quality assessment. Studies were included on the basis of their relevance to the research question, adherence to eligibility criteria, and data availability.

This scoping review protocol was registered on the Open Science Framework (OSF) and is available at DOI: https://doi.org/10.17605/OSF.IO/A8W9Q.

## Results

### Selection of sources of evidence

Most of the included studies were retrospective cohort studies (*n* = 12), post hoc analyses (*n* = 8), one cross-sectional study (*n* = 1), and one self-controlled sequence symmetry analysis (*n* = 1). These studies reported their results using descriptive statistics and involved different sample sizes. The publications ranged from 2018 to 2024 and were conducted in the United Kingdom (*n* = 5), Canada (*n* = 4), the United States (*n* = 3), Japan (*n* = 3), China (*n* = 2), Germany (*n* = 2), Taiwan (*n* = 1), Denmark (*n* = 1), and France (n=1). The mean follow-up time ranged from 0.8 to 6 years. Study characteristics—authors, country, journal, design, population, sample, and follow-up—are available in Additional file 3.

**Table 1:** SGLT2i types, comparators, outcomes, and main findings

Study	Author and year	SGLT2i	Comparator	Outcomes analyzed	Main findings
S1	Ouchi et al., 2018 [28]	Tofogliflozin	Placebo	Decrease in SUA concerning quartiles of glycated Hb	Significant ↓ SUA, especially in patients with HbA1c ≤ 7.4% (quartile 1); greatest ↓ in the first 4 weeks (approximately 0.8 mg/dL).
S2	Li et al., 2019 [29]	Canagliflozin	Placebo	Gout flares or starting a gout medication	6.7% ↓ SUA (−23.3 μmol/L, 95% CI −25.4 to −21.3) and ↓ gout flares or need for gout treatment in the canagliflozin vs. placebo group (HR 0.53, 95% CI 0.40–0.71; *p* < 0.0001).
S3	Fralick et al., 2020 [30]	Any	GLP1-RA	Incidence of gout	↓ incidence of gout in SGLT2i users (HR 0.64 [95% CI, 0.57 to 0.72]) and rate difference of −2.9 (95% CI, −3.6 to −2.1) per 1000 PY.
S4	Chung et al., 2021 [31]	Canagliflozin Dapagliflozin Empagliflozin	DPP4i	Incidence of gout	↓ risk of gout (HR, 0.89; 95% CI, 0.82- 0.96) in the SGLT2i group (already adjusted for risk factors).
S5	Lund et al., 2021 [32]	Any	GLP1-RA	Incidence of gout	↓ incidence of gout in the SGLT2i group (difference rate of −3.0 [95% CI: −4.4 to − 1.5] per 1,000 PY and HR of 0.58 [0.44 to 0.75])
S6	Doehner et al., 2022 [33]	Empagliflozin	Placebo	Association between SUA and the primary composite outcome of CV death or hospitalization for worsening HF, its components, and all- cause mortality.	Elevated SUA (highest tertile, mean SUA 9.38 ± 1.49 mg/dL) were associated with advanced HF severity and worse outcomes (composite outcome, HR 1.64 [95% CI, 1.28–2.10]; CV mortality, HR 1.98 [95% CI 1.35–2.91]; all-cause mortality, HR 1.8 [95% CI 1.29–2.49], all *p* < 0.001), compared with the lowest SUA tertile; SUA was reduced after empagliflozin treatment at 4 weeks (vs. placebo: −1.12 ± 0.04 mg/dL, *p* = 0.0001) and remained lower throughout follow-up. Empagliflozin ↓ events of acute gout, gouty arthritis, or initiation of anti-gout therapy by 32% (HR 0.68 [95% CI 0.52–0.89], *p* = 0.004); The beneficial effect of empagliflozin on the primary outcome was independent of baseline SUA (HR 0.76 [95% CI 0.65–0.88], *p* = 0.001) and change in SUA at 4 weeks (HR 0.81 [95% CI 0.69–0.95], *p* = 0.012).
S7	Ferreira et al., 2022 [34]	Empagliflozin	Placebo	Composite outcome of new prescription of anti-gout medications or gout episodes.	Adjusted mean difference reduction in SUA at week 52 = − 0.37 mg/dL (95% CI, −0.42, −0.31) and composite endpoint (HR 0.67 [95% CI 0.53, 0.85; *p* = 0.001]) in the empagliflozin group (10 or 25 mg).
S8	Gouda et al., 2022 [35]	Any	Other OAD	Need to start anti- gout/anti-hyperuricemic medications	SGLT2i group: less need to initiate anti- gout/ anti-hyperuricemic treatment (RR 0.37; 95% CI 0.20–0.68; *p* < 0.001).
S9	McDowell et al., 2022 [36]	Dapagliflozin (tertile 3)	Dapagliflozin (tertile 1) or placebo	Association between SUA and primary composite outcome of CV death or worsening HF, its components, and all- cause mortality, comparing patients by SUA tertiles [tertile 3 (SUA ≥ 6.8 mg/dL) versus tertile 1 (<5.4 mg/dL)], after adjustment for other prognostic variables; Change in SUA from baseline over 12 months	Higher SUA were associated with a higher risk of the primary outcome (adjusted HR tertile 3 vs. tertile 1: 1.32, 95% CI 1.06–1.66; *p* = 0.01). The risk of HF hospitalization and CV death increased by 7 and 6%, respectively, per 1 mg/dl increase in SUA (*p* = 0.04 and *p* = 0.07). SUA reduced by 0.84 mg/dl (95% CI −0.93 to −0.74) over 12 months (*p* < 0.001) in the dapagliflozin group. Dapagliflozin improved outcomes regardless of baseline SUA concentration.
S10	Butt et al., 2023 [37]	Dapagliflozin	Placebo	Primary outcome: composite of worsening HF or CV death. Other clinical outcomes were: Hospitalization for HF or CV death; Worsening HF; Hospitalization for HF; CV death; All- cause death; Total hospitalizations for HF and CV death. Analyzed the association between gout and clinical outcomes and the effect of dapagliflozin in patients with and without gout, and the introduction of a new uric acid-lowering therapy and colchicine (as a surrogate for gout flares).	Patients with gout vs without gout showed a greater association with the following outcomes: Worsening of HF or CV death (HR 1.15 [1.01–1.31] 95% CI); Hospitalization for HF or CV death (HR 1.16 [1.01–1.32] 95% CI); Worsening of HF (HR 1.19 [1.02–1.38] 95% CI); Hospitalization for HF (HR 1.19 [1.01- 1.39] 95% CI) already corrected for the various risk and demographic factors. Compared with placebo, dapagliflozin reduced the risk of worsening HF or CV death (primary outcome) by the same amount in patients with gout (HR, 0.84; 95% CI, 0.66–1.06) and without gout (HR, 0.79; 95% CI, 0.71–0.87). Dapagliflozin was also associated with a reduced risk of starting a uric acid–lowering therapy (HR, 0.43; 95% CI, 0.34–0.53) or colchicine (HR, 0.54; 95% CI, 0.37–0.80) vs. placebo.
	McCormick et al., 2023 [38]	Any	DPP4i	Gout flares (determined by Emergency room hospitalizations, outpatient, and medication dispensing records); secondary outcomes: HA and stroke.	Gout flares rate was lower among SGLT2i initiators RR 0.66 (95% CI, 0.57 to 0.75) and RD, −27.4 (CI, −36.0 to −18.7) per 1000 PY; RR and RD for emergency room visits and hospitalizations for gout were 0.52 (CI, 0.32 to 0.84) and −3.4 (CI, −5.8 to −0.9) per 1000 PY, respectively. The corresponding HR and RD for HA were 0.69 (CI, 0.54 to 0.88) and −7.6 (CI, −12.4 to −2.8) per 1000 PY; the HR for stroke was 0.81 (CI, 0.62 to 1.05).
S12	Subramanian et al., 2023 [39]	Any	DPP4i	Incidence of gout	In the adjusted model, the adjusted HR was 1.3 (95% CI 0.90–2.29). Sensitivity analyses did not alter the findings.
S13	Wei et al., 2023 [40]	Any	GLP1-RA or DPP4i	Primary outcome: number of recurrent gout flares; secondary outcomes: first recurrent gout flare and all-cause mortality.	The relative rate of recurrent flares with SGLT2i vs. active comparators was 0.79 (95% CI, 0.65–0.97). For the first recurrent flare with SGLT2i vs. active comparators, the RD was −8.8 (95% CI, −17.2 to −0.4) per 1000 PY and HR 0.81 (95% CI, 0.65–0.98). All-cause mortality per 1000 PY was 18.8 for SGLT2i and 24.9 for active comparators, with an RD of −6.1 (95% CI, − 10.6 to −1.6) per 1000 PY and HR 0.71 (95% CI, 0.52–0.97).
S14	Wood et al., 2023 [41]	Any	GLP1-RA, DPP4i or glitazones	Comparison of gout incidence before and after initiation of SGLT2i.	↓ gout incidence (SR = 0.62; 95% CI: 0.53–0.72) after SGLT2i use. A ↓ in gout incidence was also observed in exposure counterfactual cohorts initiating DPP4i (SR = 0.67; 95% CI: 0.63–0.72) and thiazolidinediones (SR = 0.72; 95% CI: 0.65–0.79), but not with GLP1-RA (SR = 0.93; 95% CI: 0.77–1.12).
S15	Zhou et al., 2023 [23]	Any	DPP4i	Incidence of gout; all- cause mortality.	SGLT2I were associated with 51% ↓ risks of gout (HR: 0.49; 95% CI: 0.42, 0.58; *p* < 0.0001) and 51% ↓ risks of all-cause mortality (HR: 0.49; 95% CI: 0.42, 0.58; *p* < 0.0001) after adjustment for significant demographics, prior comorbidities, medications, and laboratory results.
S16	Doehner et al., 2024 [42]	Empagliflozin	Placebo	Clinical effect of SUA reduction on the primary composite outcome of CV mortality or HF hospitalization, their individual components, and all- cause mortality in patients with HFpEF.	Elevated SUA (highest tertile of SUA 8.8 ± 1.4 g/dL) were associated with advanced HF severity and higher risk of adverse outcomes (primary outcome HR: 1.23 [95% CI: 0.98–1.53]; *p* = 0.07; HF hospitalization HR: 1.42 [95% CI: 1.08–1.86]; *p* = 0.01); SUA was reduced early (after 4 weeks vs. placebo − 0.99 +/- 0.03 mg/dL; *p* < 0.0001) and throughout follow-up, with ↓ in all prespecified subgroups. Empagliflozin ↓ clinical events of hyperuricemia (acute gout, gouty arthritis, or initiation of antigout therapy) by 38% (HR: 0.62 [95% CI: 0.51–0.76]; *p* < 0.0001). Treatment benefit on the primary outcome was not influenced by baseline SUA (HR: 0.79 [95% CI: 0.69–0.90]; *p* = 0.0004).
S17	McCormick et al., 2024 [43]	Any	GLP1-RA or DPP4i	Nephrolithiasis resulting in hospitalization or emergency room visits and gout flare- ups.	SGLT2i were associated with a lower rate of gout flares (rate ratio of 0.72, 0.54 to 0.95, rate difference of −16, −31 to −1 per 1000 PY) versus GLP1-RA (0.65, 0.52 to 0.82 and −21, −33 to −9 per 1000 PY) versus DPP4i.
S18	McCormick et al., 2024 [44]	Any	Sulfonylureas	Primary outcome: Incident gout; Secondary outcome: primary gout-related hospitalization and ER visits, and MACE, as well as flare recurrence rates among patients with prior gout.	The SGLT2i group had a lower incidence of gout (RR, 0.62 [95% CI, 0.48–0.80]) and RD, −2.64 (95% CI, −3.99 to −1.29) per 1000 PY. (regardless of sex, age, or baseline diuretic use) and fewer recurrent flares among patients with gout (RR, 0.67; 95% CI, 0.55–0.82; and RD, −20.9; 95% CI, −31.9 to −10.0 per 1000 PY). HR and RD for MACE associated with SGLT2i use were 0.87 (95% CI, 0.77–0.98) and −3.58 (95% CI, −6.19 to −0.96) per 1000 PY.
S19	Preston et al., 2024 [45]	Any	GLP1-RA; Metformin or insulin alone	Incidence of gout, all- cause mortality (positive control), and herpes zoster infection (negative control)	↓ incidence of gout at 5 years in the SGLT2i plus metformin vs. metformin (HR 0.75 [95% CI 0.69–0.82], *p* < 0.0001) and the comparison SGLT2i plus insulin vs. insulin (HR 0.83 [95% CI 0.74–0.92], *p* < 0.0001). No significant disparity in the incidence of gout was observed between GLP-1RA and matched controls. Subgroup analysis demonstrated an ↓ incidence of gout with SGLT2i vs. GLP-1RA, in groups that used metformin (HR 0.77 [95% CI 0.70–0.86], *p* < 0.0001) or insulin (HR 0.82 [95% CI 0.73–0.91], *p* < 0.0001).
S20	Sridhar et al., 2024 [46]	Ertugliflozin	Placebo	Composite outcome of incidence of new cases of gout or initiation of anti-gout medication; ↓ SUA	The incidence of the composite endpoint was 3.3% for placebo and 2.6% for ertugliflozin (HR 0.76 [95% CI 0.580, 1.002]). Ertugliflozin were associated with ↓ SUA across all SUA quintiles from baseline compared with placebo.
S21	Yokose et al., 2024 [47]	Canagliflozin Dapagliflozin Empagliflozin	Sulfonylureas	↓ SUA with SGLT2i in patients with and without T2DM; comparison of SUA changes between SGLT2i and sulfonylurea.	Among SGLT2i initiators, the within-group mean change in SUA was −1.8 (95% CI, − 2.4 to −1.1) mg/dL, including −1.2 (−1.8 to − 0.6) mg/dL and −2.5 (−3.6 to −1.3) mg/dL among patients with and without T2DM, respectively, with an adjusted difference between those with and without T2DM of − 1.4 (−2.4 to −0.5) mg/dL; SUA did not change after sulfonylurea initiation (+0.3 [− 0.3 to 1.0] mg/dL). The adjusted difference in SUA between SGLT2i initiation and sulfonylurea initiation was −1.8 (−2.7 to − 0.9) mg/dL in all patients.
S22	Yokoyama et al., 2024 [48]	Any	Self-controlled SSA	Initiation of a hypouricemic agent or incidence of gout	Trend-adjusted sequence ratios were calculated at 6, 12, 18, and 24-month intervals. Significant inverse signals were observed at all intervals between SGLT2i and hypouricemic agents, with the strongest effect observed at the 24-month interval (adjusted sequence ratio 0.52 [95% CI 0.49–0.55]). Significant inverse signals were observed for each of the six types of SGLT2i at all intervals.

**Abbreviations:** SGLT2i - Sodium-glucose cotransporter 2 inhibitor; GLP1-RA - Glucagon like peptide-1 receptor agonists; DPP4i - dipeptidyl peptidase-4 inhibitors; OAD - Oral antidiabetic drugs (sulfonylureas, biguanides, thiazolidinediones, a-glucosidase inhibitors, glinides and DPP4i); SSA - Sequence symmetry analysis; SUA - serum uric acid; P - *p*-value; PY - Person-Years; HR - Hazard ratio; CI - Confidence interval; RR - Rate ratio; RD - Rate difference; SR - Symmetry ratio; CV - Cardiovascular; HF - Heart failure; HA - Heart attack; HFpEF - Heart failure with preserved ejection fraction; ER - Emergency room; MACE - Major adverse cardiovascular events

**Table 2:** Outcomes of interest evaluated in the studies and the number of related studies

Outcomes	Studies	Nº
Reduction in serum uric acid	S1, S2, S6, S7, S9, S16, S20, S21	8
Decreased incidence of gout	S3, S4, S5, S6, S7, S12*, S14, S15, S18, S19, S20, S22	12
Reduction in gout attacks	S2, S6, S7, S11, S13, S16, S17, S18	8
Reduction in the prescription of a new uric acid-lowering therapy or colchicine	S2, S6, S7, S8, S10, S16, S20, S22	8
Decreased emergency room visit rates and/or hospitalization rates for gout	S11	1
Decrease in all-cause mortality	S6, S9, S13, S15, S16	5
Decrease in cardiovascular mortality	S6, S9, S10, S16	4

*S12: No statistical difference. Neutral effects of SGLT2i

### Characteristics of the studied sample

The mean age of the participants ranged from 52 to 72 years, with a higher prevalence of male individuals (*n* = 21). Most samples included predominantly White (n =11) or Asian (*n* = 5) participants. There was considerable variation in the percentage of individuals with gout at baseline among the studies. In three studies, 100% of the patients had gout. In seven studies, this information was not reported. In the remaining studies, the proportion of individuals with gout at baseline ranged from 0% to 22%. For CV comorbidities and CKD, these conditions were highly prevalent across the studies, with only three studies not reporting such data. Baseline characteristics—age, sex, ethnicity, gout, cardiovascular comorbidities, and CKD—are available in Additional file 4.

### Results of individual sources of evidence and synthesis of the results

Among the studies included in this review, the most frequently used comparators were placebo (*n* = 7), dipeptidyl peptidase-4 inhibitors (DPP4i) (*n* = 4), glucagon-like peptide-1 receptor agonists (GLP1-RA) (*n* = 2), GLP1-RA or DPP4i (*n* = 2), sulfonylureas (*n* = 2), and other comparator groups (*n* = 5).

Regarding the outcomes of interest, most studies reported data on the reduction in gout incidence (*n* = 12), SUA levels (*n* = 8), gout flares (*n* = 8), and the prescription of new urate-lowering therapy or colchicine (*n* = 8). Only one study analyzed the reduction in emergency room visits and/or hospitalizations related to gout among SGLT2i users.

Table 1 presents the types of SGLT2i used, comparators, analyzed outcomes, and main findings of each study. Table 2 lists the outcomes of interest addressed in the studies and the number of studies related to each outcome.

## Discussion

### Summary of evidence

Overall, SGLT2i were associated with several relevant clinical and laboratory effects in patients with T2DM and gout. In this review, among the 22 analyzed studies, SGLT2i were associated with beneficial effects on the following outcomes: a reduction in SUA, a decreased incidence of gout, a reduction in gout flares, a reduced prescription of new uric acid-lowering therapies or colchicine, decreased emergency room visits and/or hospitalization rates due to gout, and reductions in both all-cause and CV-related mortality. These findings were consistent across different SGLT2i types, suggesting a potential class rather than a drug-specific effect. It is important to note that the majority of the included studies were retrospective in design, which limits the ability to infer causality from the observed associations.

Mechanistically, SGLT2i promote uricosuria through glycosuria, which competes with urate for reabsorption in the proximal tubule, thereby increasing urinary urate excretion and lowering SUA levels. Several meta-analyses [49, 50] have reported a mean reduction in SUA of approximately 0.6 mg/dL, notably in patients using dapagliflozin and empagliflozin [49–51]. This urate-lowering effect appears to be independent of baseline SUA, diuretic use, or other metabolic variables [49].

In this review, all eight studies that assessed SUA reduction reported benefits associated with SGLT2i use (Tables 1 and 2) compared with respective comparators (placebo or sulfonylureas), with statistical significance. SUA reductions ranged from 0.19 mg/dL with ertugliflozin [46] to 1.8 mg/dL in a study involving canagliflozin, dapagliflozin, or empagliflozin as interventions [47]. However, these results varied across studies evaluating the same drugs. For example, empagliflozin led to a 0.37 mg/dL reduction in the study by Ferreira et al. [34], and canagliflozin reduced SUA by 0.39 mg/dL in the study by Li et al. [29], which contrasts with the 1.8 mg/dL reduction reported by Yokose et al. [47]. Methodological and clinical differences between studies explain the variation in the magnitude of urate reduction. Factors such as study design, baseline sample profile, comparator drug, and follow-up time, as well as the heterogeneity of interventions and outcome metrics, justify these disparities.

In addition to lowering urate levels, SGLT2i may exert anti-inflammatory effects relevant to gout pathophysiology, including the downregulation of IL-1β, IL-6, TNF-α, and MCP-1, as well as a reduction in C-reactive protein levels. These pleiotropic effects may contribute to decreased gout flares and disease severity, independent of SUA changes [44, 52]. In this review, SGLT2i use was consistently associated with benefits in all studies that assessed gout flares (*n* = 8) and in those evaluating the prescription of anti-gout medications (*n* = 8), as presented in Tables 1 and 2. However, outcomes such as the initiation of urate-lowering therapy or colchicine should be interpreted as surrogate markers, as they reflect clinical decision-making rather than direct measures of disease activity or flare severity. The comparators included placebo, GLP1-RA, DPP4i, sulfonylureas, and other oral antidiabetic drugs, such as biguanides, thiazolidinediones, α-glucosidase inhibitors, and glinides.

A critical limitation of the current evidence base is that only three of the 22 included studies evaluated exclusively patients with both T2DM and established gout [38, 40, 47]. All were retrospective cohort studies. This considerably limits the generalizability of our findings to the target population of interest.

McCormick et al. [38] analyzed 8,150 patients over a mean follow-up of 1.6 years and compared different SGLT2i with DPP4i. They reported a reduction in gout flares (rate ratio [RR] 0.66; 95% confidence interval [CI], 0.57 to 0.75, and rate difference [RD] −27.4 [CI, −36.0 to −18.7] per 1000 person-years [PY]), a decrease in emergency visits or hospitalizations due to gout (RR 0.52 [CI, 0.32 to 0.84] and RD −3.4 [CI, −5.8 to − 0.9] per 1000 PY), and a reduction in HA (hazard ratio [HR] 0.69 [CI, 0.54 to 0.88]). Stroke risk was also reduced (HR 0.81; 95% CI: 0.62 to 1.05), although this difference was not statistically significant.

Wei et al. [40] analyzed 5,931 patients over a mean follow-up of 2.7 years and compared SGLT2i users with those using GLP1-RA or DPP4i. The authors reported statistically significant reductions in gout flares (RR 0.79; 95% CI: 0.65 to 0.97) and all-cause mortality (HR 0.71; 95% CI: 0.52 to 0.97).

Yokose et al. [47] analyzed 56 patients treated with SGLT2i or sulfonylureas for a mean of 6 months. They reported SUA reductions in patients with and without T2DM (mean change: −1.8 mg/dL; 95% CI: −2.4 to −1.1), with more pronounced effects in those without T2DM (−2.5 mg/dL; 95% CI: −3.6 to −1.3). The sulfonylurea group showed no change in SUA.

Regarding the incidence of gout, 11 out of the 12 studies evaluating this outcome demonstrated beneficial effects of SGLT2i. The exception was the study by Subramanian et al. [39], which included 43,043 patients with T2DM and new users of SGLT2i or DPP4i (follow-up of 1.7 years), without finding a significant difference. The remaining studies demonstrated positive results compared with placebo [33, 34, 46], DPP4i [23, 31], GLP1-RA [30, 32], DPP4i or GLP1-RA or glitazones [41], sulfonylureas [44], GLP1-RA or metformin or insulin [45], and self-controlled sequence symmetry analysis (SSA) [48], further supporting the potential role of SGLT2i in reducing the risk of gout onset.

However, the discrepancy between the study by Subramanian et al. [39] and the others should be interpreted with caution due to methodological limitations that compromised the analytical sensitivity of this study. First, the short median follow-up period (1.7 years) and the low rate of incident events (*n* = 85) resulted in reduced statistical power to detect differences between active comparators. A determining factor, identified in the detailed analysis of the baseline characterization, is the significant incompleteness of the serum uricemia data, with approximately 85% of the sample classified as missing data. This gap makes it impossible to confirm the comparability between the groups regarding the main risk factor for the outcome and suggests the persistence of unmeasured residual confounding. Therefore, the neutrality of this study seems to derive from weaknesses in the quality of real-world data and an insufficient observation window, not invalidating the protective effect of SGLT2i observed in the other studies.

Only one study [38] specifically evaluated reductions in emergency visits and/or hospitalizations due to gout, highlighting a gap and an opportunity for future research. This study reported RR and RD values of 0.52 (95% CI: 0.32 to 0.84) and −3.4 (95% CI: − 5.8 to −0.9) per 1000 PY, respectively.

In addition, SGLT2i have been associated with CV and renal benefits, which are especially relevant in gout patients because of their high cardiometabolic burden [21, 52]. The most robust data regarding all-cause and CV-related mortality come from post hoc analyses of major clinical trials. Doehner et al. [33] conducted a post hoc analysis of the EMPEROR-Reduced trial, which evaluated empagliflozin in 3,676 patients with HF with reduced ejection fraction (HFrEF), with or without T2DM, over a mean follow-up of 1.3 years. The percentage of patients with gout was not reported, but 53% of the sample had hyperuricemia. The main findings were that higher SUA levels were associated with worse outcomes. Specifically, patients in the highest SUA tertile (mean 9.38 ± 1.49 mg/dL) had significantly greater risks of CV death or hospitalization for HF (HR 1.64; CI: 1.28 to 2.10), CV mortality (HR 1.98; CI: 1.35 to 2.91), and all-cause mortality (HR 1.80; CI: 1.29 to 2.49).

Importantly, empagliflozin improved the primary endpoint regardless of baseline SUA (HR 0.76; 95% CI: 0.65–0.88; *p* = 0.001) or SUA reduction at four weeks (HR 0.81; 95% CI: 0.69 to 0.95; *p* = 0.012).

Several post hoc analyses—DAPA-HF [36], DAPA-HF and DELIVER [37], and EMPEROR-Preserved [42]—demonstrated similar results in all-cause and CV-related mortality, even after multivariate-adjusted analyses. All the studies used placebo as a comparator, and the associations of SGLT2i with better outcomes were independent of SUA levels and gout status. Two retrospective cohort studies also evaluated mortality. As previously discussed, Wei et al. [40] reported lower all-cause mortality in 5,931 patients (HR 0.71; CI: 0.52 to 0.97). Zhou et al. [23], in a cohort of 43,201 patients with T2DM (mean follow-up: 5.6 years), reported a 51% reduction in all-cause mortality among SGLT2i users compared with DPP4i users (HR 0.49; CI: 0.42 to 0.58; *p* < 0.0001), even after adjusting for demographics, comorbidities, medications, and laboratory values.

The focus of this scoping review was to analyze the clinical and laboratory outcomes of SGLT2i specifically in patients with both T2DM and gout. However, only three studies included samples composed entirely of patients with both conditions [38, 40, 47]. In the remaining studies, the proportion of patients with gout ranged from 5% [29] to 22% [43], and in some, these data were not clearly stated.

We consider this a window of opportunity for future studies focused specifically on this subpopulation. Conversely, when evaluating gout incidence, it is necessary to exclude patients with gout at baseline to assess the occurrence of new cases during follow-up. Twelve studies in this review analyzed this outcome (Table 2).

Among the 22 studies included, SGLT2i were frequently associated with favorable effects on the analyzed outcomes.

### Limitations

As this was a scoping review, we did not perform a risk of bias assessment of the included studies. Furthermore, several studies failed to report clearly the proportion of patients with gout in their samples—a detail of great relevance for the scope of this review.

Another limitation is the predominance of retrospective cohort studies. This design is associated with a higher risk of bias, limited ability to establish causality, less precise temporality, and dependence on historical medical records.

## Conclusion

The findings of this scoping review suggest favorable associations of SGLT2i in patients with gout and T2DM, including improvements in gout-related clinical, laboratory, and CV outcomes. Given the high prevalence of T2DM and the multiple components of metabolic syndrome commonly observed in patients with gout—as well as the recognition of gout as an independent CV risk factor—SGLT2i may have potential therapeutic relevance for this subpopulation, potentially contributing to the management of both conditions and the reduction of associated CV events. However, prospective studies specifically including patients with both T2DM and established gout are warranted before definitive clinical recommendations can be made.

## Electronic supplementary material

Below is the link to the electronic supplementary material.

Supplementary Material 1: Additional file 1

Supplementary Material 2: Additional file 2

Supplementary Material 3: Additional file 3

Supplementary Material 4: Additional file 4

## Data Availability

All data generated or analyzed during this study are included in this published article and its supplementary information files (Additional file 1: PRISMA-ScR checklist; Additional file 2: Search strategy).

## Abbreviations

CAD: Coronary artery disease
CI: Confidence interval
CKD: Chronic kidney disease
CV: Cardiovascular
CVD: Cardiovascular disease
DLP: Dyslipidemia
DM: Diabetes mellitus
DPP4i: Dipeptidyl peptidase-4 inhibitors
ER: Emergency room
GLP1-RA: Glucagon-like peptide-1 receptor agonists
HA: Heart attack
HF: Heart failure
HFpEF: Heart failure with preserved ejection fraction
HFrEF: Heart failure with reduced ejection fraction
HR: Hazard ratio
HTN: Hypertension
MACE: Major adverse cardiovascular events
OAD: Oral antidiabetic drugs (sulfonylureas, biguanides, thiazolidinediones, α- glucosidase inhibitors, glinides, and DPP4i)
p: *p*-value
PY: Person-years
RD: Rate difference
RR: Rate ratio
SGLT2i: Sodium-glucose cotransporter 2 inhibitors
SR: Symmetry ratio
SSA: Sequence symmetry analysis
SUA: Serum uric acid
T2DM: Type 2 diabetes mellitus
ULT: Urate-lowering therapy

## Declarations

### Authors’ information

HPS is the current coordinator of the Microcrystalline Arthropathies Committee of the Brazilian Society of Rheumatology, with a special interest in gout and microcrystalline arthropathies. BCHS is a preceptor at the Bauru Diabetes Outpatient Clinic, Bauru School of Medicine, University of São Paulo (FMBRU-USP). CAN is a member of the Department of Diabetes in Pregnancy of the Brazilian Diabetes Society.

## References

[1] International Diabetes Federation. IDF Diabetes Atlas. 2025. https://diabetesatlas.org/resources/idf-diabetes-atlas-2025/. 11th ed. Brussels: IDF; 2025. Available from.

[2] Lim LL, Chow E, Chan C. Cardiorenal diseases in type 2 diabetes mellitus: clinical trials and real-world practice. Nat Rev Endocrinol. 2023;19(3):151–63. 10.1038/s41574-022-00776-2. 36446898

[3] Dalbeth N, Choi HK, Joosten LAB, Khanna PP, Matsuo H, Perez-Ruiz F, et al. Gout (primer). Nat Rev Dis Primers. 2019;5(1):69. 10.1038/s41572-019-0115-y. 31558729

[4] Dalbeth N, Gosling AL, Gaffo A, Abhishek AG. Gout. Lancet. 2021;397(10287):1843–55. 10.1016/S0140-6736(21)00569-9. 33798500

[5] Loeb JN. The influence of temperature on the solubility of monosodium urate. Arthritis Rheumatism. 1972;15(2):189–92. 10.1002/art.1780150209. 5027604

[6] Campion EW, Glynn RJ, DeLabry LO. Asymptomatic hyperuricemia. Risks and consequences in the normative aging study. Am J Med. 1987;82(3):421–26. 10.1016/0002-9343(87)90441-4. 3826098

[7] Dalbeth N, Phipps-Green A, Frampton C, Neogi T, Taylor WJ, Merriman TR. Relationship between serum urate concentration and clinically evident incident gout: an individual participant data analysis. Ann Rheumatic Dis. 2018;77(7):1048–52. 10.1136/annrheumdis-2017-212288. 29463518

[8] Dehlin M, Jacobsson L, Roddy E. Global epidemiology of gout: prevalence, incidence, treatment patterns and risk factors. Nat Rev Rheumatol. 2020;16(7):380–90. 10.1038/s41584-020-0441-1. 32541923

[9] Dório M, Benseñor, Lotufo P, Santos IS, Fuller R, Benseñor IM. Reference range of serum uric acid and prevalence of hyperuricemia: a cross-sectional study from baseline data of ELSA-Brasil cohort. Adv Rheumatol. 2022;62(1):15. 10.1186/s42358-022-00246-3. 35527261

[10] Zhu Y, Pandya BJ, Choi HK. Comorbidities of gout and hyperuricemia in the US general population: NHANES 2007-2008. Am J Med. 2012;125(7):679–87.e1. 10.1016/j.amjmed.2011.09.033. 22626509

[11] Choi HK, Ford ES, Li C, Curhan G. Prevalence of the metabolic syndrome in patients with gout: the third national health and nutrition examination survey. Arthritis Rheumatism. 2007;57(1):109–15. 10.1002/art.22466. 17266099

[12] Singh JA, Cleveland JD. Gout and the risk of myocardial infarction in older adults: a study of Medicare recipients. Arthritis Res Ther. 2018;20(1):109. 10.1186/s13075-018-1606-z. PMC5984737 29859125

[13] Cox P, Gupta S, Zhao SS, Hughes DM. The incidence and prevalence of cardiovascular diseases in gout: a systematic review and meta-analysis. Rheumatol Int. 2021;41(7):1209–19. 10.1007/s00296-021-04876-6. PMC8164620 33987709

[14] Choi HK, Curhan G. Independent impact of gout on mortality and risk for coronary heart disease. Circulation. 2007;116(8):894–900. 10.1161/CIRCULATIONAHA.107.703389. 17698728

[15] Teng GG, Ang LW, Saag KG, Yu MC, Yuan JM, Koh WP. Mortality due to coronary heart disease and kidney disease among middle-aged and elderly men and women with gout in the Singapore Chinese health study. Ann Rheumatic Dis. 2012;71(6):924–28. 10.1136/ard.2011.200523. PMC3400339 22172492

[16] Kuo CF, See LC, Luo SF, Ko YS, Lin YS, Hwang JS, et al. Gout: an independent risk factor for all-cause and cardiovascular mortality. Rheumatology. 2010;49(1):141–46. 10.1093/rheumatology/kep364. 19933595

[17] Chen JH, Chuang SY, Chen HJ, Yeh WT, Pan WH. Serum uric acid level as an independent risk factor for all-cause, cardiovascular, and ischemic stroke mortality: a Chinese cohort study. Arthritis Rheumatism. 2009;61(2):225–32. 10.1002/art.24164. 19177541

[18] Cipolletta E, Tata LJ, Nakafero G, Avery AJ, Mamas MA, Abhishek A. Association between gout flare and subsequent cardiovascular events among patients with gout. JAMA. 2022;328(5):440–. 10.1001/jama.2022.11390. PMC9346550 35916846

[19] Choi HK, McCormick N. Beyond joint pain, could each gout flare lead to heart attack? Nat Rev Rheumatol. 2022;18(11):619–20. 10.1038/s41584-022-00844-x. 36131085

[20] Idris I, Donnelly R. Sodium–glucose co-transporter-2 inhibitors: an emerging new class of oral antidiabetic drug. Diabetes Obes Metab. 2009;11(2):79–88. 10.1111/j.1463-1326.2008.00982.x. 19125776

[21] Kochanowska A, Rusztyn P, Szczerkowska K, Surma S, Gąsecka A, Jaguszewski MJ, et al. Sodium–glucose cotransporter 2 inhibitors to Decrease the uric acid concentration—A novel mechanism of action. J Cardiovasc Dev Dis. 2023;10(7):268. 10.3390/jcdd10070268. PMC10380892 37504524

[22] Zhao Y, Xu L, Tian D, Xia P, Zheng H, Wang L, et al. Effects of sodium-glucose co-transporter 2 (SGLT2) inhibitors on serum uric acid level: a meta-analysis of randomized controlled trials. Diabetes Obes Metab. 2018;20(2):458–62. 10.1111/dom.13101. 28846182

[23] Zhou J, Liu X, Chou OH-I, Li L, Lee S, Wong WT, et al. Lower risk of gout in sodium glucose cotransporter 2 (SGLT2) inhibitors versus dipeptidyl peptidase-4 (DPP4) inhibitors in type-2 diabetes. Rheumatology. 2023;62(4):1501–10. 10.1093/rheumatology/keac509. 36066415

[24] Banerjee M, Pal R, Mukhopadhyay S. Can SGLT2 inhibitors prevent incident gout? A systematic review and meta-analysis. Acta Diabetol. 2022;59(6):783–91. 10.1007/s00592-022-01866-3. 35249140

[25] Lai SW, Hwang BF, Kuo YH, Liu CS, Liao KF. Sodium-glucose cotransporter-2 inhibitors use and the risk of gout: a systematic review and meta-analysis. Front Endocrinol. 2023;14:1158153. 10.3389/fendo.2023.1158153. PMC10242385 37288295

[26] Peters MDJ, Godfrey C, McInerney P, Munn Z, Tricco AC, Khalil H. Scoping reviews. In: Aromataris E, Lockwood C, Porritt K, Pilla B, Jordan Z, editors. JBI manual for evidence synthesis. JBI; 2024. Available from: 10.46658/JBIMES-24-09.

[27] Tricco AC, Lillie E, Zarin W, O’Brien KK, Colquhoun H, Levac D, et al. PRISMA extension for scoping reviews (PRISMA-ScR): checklist and explanation. Ann Intern Med. 2018;169(7):467–73. 10.7326/M18-0850. 30178033

[28] Ouchi M, Oba K, Kaku K, Suganami H, Yoshida A, Fukunaka Y, et al. Uric acid lowering in relation to HbA1c reductions with the SGLT2 inhibitor tofogliflozin. Diabetes Obes Metab. 2018;20(4):1061–65. 10.1111/dom.13170. PMC5887894 29171930

[29] Li J, Badve SV, Zhou Z, Rodgers A, Day R, Oh R, et al. The effects of canagliflozin on gout in type 2 diabetes: a post-hoc analysis of the CANVAS Program. The Lancet Rheumatol. 2019;1(4):e220–28. 10.1016/S2665-9913(19)30078-5. 38229378

[30] Fralick M, Chen SK, Patorno E, Kim SC. Assessing the risk for gout with sodium–glucose cotransporter-2 inhibitors in patients with type 2 diabetes. Ann Intern Med. 2020;172(3):186–94. 10.7326/M19-2610. PMC7217750 31931526

[31] Chung MC, Hung PH, Hsiao PJ, Wu LY, Chang CH, Wu MJ, et al. Association of sodium-glucose transport protein 2 inhibitor use for type 2 diabetes and incidence of gout in Taiwan. JAMA Netw Open. 2021;4(11):e2135353. 10.1001/jamanetworkopen.2021.35353. PMC8605485 34797368

[32] Lund LC, Højlund, Henriksen DP, Hallas J, Kristensen KB, Højlund M. Sodium-glucose cotransporter-2 inhibitors and the risk of gout: a Danish population based cohort study and symmetry analysis. Pharmacoepidemiol Drug. 2021;30(10):1391–95. 10.1002/pds.5252. 33881179

[33] Doehner W, Anker SD, Butler J, Zannad F, Filippatos G, Ferreira JP, et al. Uric acid and sodium- glucose cotransporter-2 inhibition with empagliflozin in heart failure with reduced ejection fraction: the EMPEROR-reduced trial. Eur Heart J. 2022;43(36):3435–46. 10.1093/eurheartj/ehac320. PMC9492270 35788657

[34] Ferreira JP, Inzucchi SE, Mattheus M, Meinicke T, Steubl D, Wanner, et al. Empagliflozin and uric acid metabolism in diabetes: a post hoc analysis of the EMPA-REG OUTCOME trial. Diabetes Obes Metab. 2022;24(1):135–41. 10.1111/dom.14559. PMC9293326 34558768

[35] Gouda M, Arakawa K, Inagaki M, Ushirogawa Y. Effect of sodium–glucose cotransporter 2 inhibitor medication on new prescriptions of antihypertensives, antigout/antihyperuricemics and antidyslipidemics in Japan: analysis using the JMDC claims database. J Diabetes Invest. 2022;13(11):1842–51. 10.1111/jdi.13887. PMC9623520 35854644

[36] McDowell K, Welsh P, Docherty KF, Morrow DA, Jhund PS, de Boer RA, et al. Dapagliflozin reduces uric acid concentration, an independent predictor of adverse outcomes in DAPA-HF. Eur J Heart Fail. 2022;24(6):1066–76. 10.1002/ejhf.2433. PMC9540869 35064721

[37] Butt JH, Docherty KF, Claggett BL, Desai AS, Petersson M, Langkilde AM, et al. Association of Dapagliflozin use with clinical outcomes and the introduction of uric acid–lowering therapy and colchicine in patients with Heart failure with and without gout. JAMA Cardiol. 2023;8(4):386–93. 10.1001/jamacardio.2022.5608. PMC9947801 36811901

[38] McCormick N, Yokose C, Wei J, Lu N, Wexler DJ, Aviña-Zubieta JA, et al. Comparative effectiveness of sodium–glucose cotransporter-2 inhibitors for recurrent gout flares and gout-primary emergency department visits and hospitalizations. Ann Intern Med. 2023;176(8):1067–80. 10.7326/M23-0724. PMC11921103 37487215

[39] Subramanian A, Gokhale K, Sainsbury C, Nirantharakumar K, Toulis KA. Sodium-glucose cotransporter-2 inhibitors and the risk of gout in patients with type 2 diabetes mellitus: a propensity-score-matched, new-user design study with an active comparator using the IQVIA Medical research data UK database. Diabetes Obes Metab. 2023;25(1):156–65. 10.1111/dom.14858. PMC10087572 36056476

[40] Wei J, Choi HK, Dalbeth N, Li X, Li C, Zeng C, et al. Gout flares and mortality after sodium- glucose cotransporter-2 inhibitor treatment for gout and type 2 diabetes. JAMA Netw Open. 2023;6(8):e2330885. 10.1001/jamanetworkopen.2023.30885. PMC10457713 37624597

[41] Wood DT, Waterbury NV, Lund BC. Sodium glucose cotransporter 2 inhibitors and gout risk: a sequence symmetry analysis. Clin Rheumatol. 2023;42(9):2469–75. 10.1007/s10067-023-06647-z. 37264145

[42] Doehner W, Anker SD, Butler J, Zannad F, Filippatos G, Coats AJS, et al. Uric acid and SGLT2 inhibition with empagliflozin in heart failure with preserved ejection fraction: the EMPEROR- preserved trial. JACC Heart Fail. 2024;12(12):2057–70. 10.1016/j.jchf.2024.08.020. 39453357

[43] McCormick N, Yokose C, Lu N, Wexler DJ, Aviña-Zubieta JA, De Vera MA, et al. Comparative effectiveness of sodium-glucose cotransporter-2 inhibitors for recurrent nephrolithiasis among patients with pre-existing nephrolithiasis or gout: target trial emulation studies. BMJ. 2024;387:80. 10.1136/bmj-2024-080035. PMC11524131 39477370

[44] McCormick N, Yokose C, Lu N, Wexler DJ, Aviña-Zubieta JA, De Vera MA, et al. Sodium-glucose cotransporter-2 inhibitors vs sulfonylureas for gout prevention among patients with type 2 diabetes receiving metformin. JAMA Intern Med. 2024;184(6):650–60. 10.1001/jamainternmed.2024.0376. PMC11019449 38619822

[45] Preston FG, Anson M, Riley DR, Ibarburu GH, Henney A, Lip GYH, et al. SGLT2 inhibitors, but not GLP-1 receptor agonists, reduce incidence of gout in people living with type 2 diabetes across the therapeutic spectrum. Clin Ther. 2024;46(11):835–40. 10.1016/j.clinthera.2024.06.021. 39068059

[46] Sridhar VS, Cosentino F, Dagogo-Jack S, McGuire DK, Pratley RE, Cater NB, et al. Effects of ertugliflozin on uric acid and gout-related outcomes in persons with type 2 diabetes and cardiovascular disease: post hoc analyses from VERTIS CV. Diabetes Obes Metab. 2024;26(11):5336–46. 10.1111/dom.15895. 39219437

[47] Yokose C, Challener G, Jiang B, Zhou B, McCormick N, Tanikella S, et al. Serum urate change among gout patients treated with sodium-glucose cotransporter type 2 inhibitors vs. sulfonylurea: a comparative effectiveness analysis. Semin Arthritis Rheumatism. 2024;66:152441. 10.1016/j.semarthrit.2024.152441. PMC11378417 38657403

[48] Yokoyama S, Nakagawa C, Uno T, Hosomi K. Evaluating the associated hyperuricemia risk with sodium-glucose cotransporter 2 inhibitors: a sequence symmetry analysis using the Japanese administrative claims database. Biol Pharm Bull. 2024;47(11):1851–57. 10.1248/bpb.b24-00330. 39522979

[49] Banerjee M, Pal R, Maisnam I, Chowdhury S, Mukhopadhyay S. Serum uric acid lowering and effects of sodium-glucose cotransporter-2 inhibitors on gout: a meta-analysis and meta-regression of randomized controlled trials. Diabetes Obes Metab. 2023;25(9):2697–703. 10.1111/dom.15157. 37334516

[50] Hu Q, Yang S, Zhang B, Su N. Effects of sodium-glucose cotransporter-2 inhibitors on serum urate levels and gout in patients with and without type 2 diabetes: a systematic review and network meta-analysis. Int J Clin Pharm. 2025;47(5):1162–76. 10.1007/s11096-025-01950-y. 40601087

[51] Mikuls TR. Gout. N Engl J Med. 2022;387(20):1877–87. 10.1056/NEJMcp2203385. PMC12939259 36383714

[52] Yokose C, McCormick N, Abhishek A, Dalbeth N, Pascart T, Lioté FGaffo A, et al. The clinical benefits of sodium–glucose cotransporter type 2 inhibitors in people with gout. Nat Rev Rheumatol. 2024;20(4):216–31. 10.1038/s41584-024-01092-x. PMC13186535 38472344

