# Supplementary material for: Effects of SGLT2 inhibitors on gout-related and cardiometabolic outcomes in patients with type 2 diabetes mellitus: a scoping review
Source: Adv Rheumatol. 2026 Jul 24;66:56. doi: 10.1186/s42358-026-00564-w (PMC13632354; doi:10.1186/s42358-026-00564-w)
Supplement: Supplementary file 2 [file 42358_2026_564_MOESM2_ESM.docx]

**Clinical and laboratory effects of sodium-glucose cotransporter 2 inhibitors in patients with type 2 diabetes mellitus and gout: a scoping review**

**Detailed database search strategy used for the scoping review across PubMed, EMBASE, Scopus, Web of Science, LILACS/BVS, BDTD, and ProQuest.**

**Additional file 1: Search strategy**

# Table 1. Search strategy and number of articles for each database, on February 7, 2025

| **Database** | | | **Search strategy** | **Nº articles** | | | | | | |
| --- | --- | --- | --- | --- | --- | --- | --- | --- | --- | --- |
| MEDLINE  PubMed | | ("adult onset diabetes mellitus"[tiab] OR "Adult-Onset Diabetes Mellitus"[tiab] OR "diabetes"[tiab] OR "DIABETES MELLITUS"[Mesh Terms] OR "DIABETES MELLITUS"[tiab] OR "diabetes mellitus type 2"[tiab] OR "diabetes mellitus type ii"[tiab] OR "DIABETES MELLITUS, TYPE 2"[Mesh Terms] OR "DIABETES MELLITUS, TYPE 2"[tiab] OR "diabetes type 2"[tiab] OR "diabetes type II"[tiab] OR "diabetic"[tiab] OR "diabets"[tiab] OR "dm 2"[tiab] OR "insulin independent diabetes"[tiab] OR "insulin independent diabetes mellitus"[tiab] OR "ketosis resistant diabetes mellitus"[tiab] OR "Ketosis-Resistant Diabetes Mellitus"[tiab] OR "Maturity Onset Diabetes"[tiab] OR "maturity onset diabetes mellitus"[tiab] OR "Maturity-Onset Diabetes"[tiab] OR "Maturity-Onset Diabetes Mellitus"[tiab] OR "MODY"[tiab] OR "NIDDM"[tiab] OR "niddm"[tiab] OR "NIDDM (non insulin dependent diabetes mellitus)"[tiab] OR "non insulin dependent (type 2) diabetes mellitus"[tiab] OR "non insulin dependent diabetes"[tiab] OR "non insulin dependent diabetes mellitus"[tiab] OR "adult onset diabetes"[tiab] OR "noninsulin dependent (type 2) diabetes mellitus"[tiab] OR "noninsulin dependent diabetes"[tiab] OR "Noninsulin Dependent Diabetes Mellitus"[tiab] OR "Noninsulin-Dependent Diabetes Mellitus"[tiab] OR "non-insulin-dependent diabetes mellitus"[tiab] OR "Slow-Onset Diabetes Mellitus"[tiab] OR "Stable Diabetes Mellitus"[tiab] OR "T2DM"[tiab] OR "TIIDM"[tiab] OR "type 2 (insulin independent) diabetes"[tiab] OR "Type 2 Diabetes"[tiab] OR "type 2 diabetes mellitus"[tiab] OR "type II diabetes"[tiab] OR "type II diabetes mellitus"[tiab] OR "unspecified diabetes mellitus"[tiab]) AND ("arthritis urica"[tiab] OR "cheiragra"[tiab] OR "chiragra"[tiab] OR "GOUT"[Mesh Terms] OR "GOUT"[tiab] OR "Gouts"[tiab] OR "Arthragra gout"[tiab] OR "gouty arthritis"[tiab] OR "gouty arthropathy"[tiab] OR "gouty attack"[tiab] OR "hyperuricaemic arthritis"[tiab] OR "hyperuricemic arthritis"[tiab] OR "urate inflammation"[tiab] OR "uric arthritis"[tiab]) AND ("Gliflozin"[tiab] OR "gliflozin derivative"[tiab] OR "Gliflozins"[tiab] OR "inhibitor sodium glucose co-transporter 2"[tiab] OR "inhibitor sodium-glucose transporter 2"[tiab] OR "inhibitors sodium dependent glucose cotransporter 2"[tiab] OR "Inhibitors sodium glucose cotransporte"[tiab] OR "SGLT 2 Inhibitor"[tiab] OR "SGLT 2 Inhibitors"[tiab] OR "SGLT2"[tiab] OR "SGLT-2 Inhibitor"[tiab] OR "SGLT-2 Inhibitors"[tiab] OR "sodium glucose cotransporter 2 inhibitor"[tiab] OR "Sodium Glucose Transporter 2 Inhibitor"[tiab] OR "Sodium Glucose Transporter 2 Inhibitors"[tiab] OR "Sodium-Glucose Transporter 2 Inhibitor"[tiab] OR "SODIUM-GLUCOSE TRANSPORTER 2 INHIBITORS"[Mesh Terms]) | | | 47 | | | | | |
| EMBASE | ("adult onset diabetes mellitus" OR "Adult-Onset Diabetes Mellitus" OR "diabetes" OR "DIABETES MELLITUS" OR "diabetes mellitus type 2" OR "diabetes mellitus type ii" OR "DIABETES MELLITUS, TYPE 2" OR "diabetes type 2" OR "diabetes type II" OR "diabetic" OR "diabets" OR "dm 2" OR "insulin independent diabetes" OR "insulin independent diabetes mellitus" OR "ketosis resistant diabetes mellitus" OR "Ketosis-Resistant Diabetes Mellitus" OR "Maturity Onset Diabetes" OR "maturity onset diabetes mellitus" OR "Maturity-Onset Diabetes" OR "Maturity-Onset Diabetes Mellitus" OR "MODY" OR "NIDDM" OR "niddm" OR "NIDDM (non insulin dependent diabetes mellitus)" OR "non insulin dependent (type 2) diabetes mellitus" OR "non insulin dependent diabetes" OR "non insulin dependent diabetes mellitus" OR "non insulin dependent diabetes mellitus" OR "adult onset diabetes" OR "noninsulin dependent (type 2) diabetes mellitus" OR "noninsulin dependent diabetes" OR "Noninsulin Dependent Diabetes Mellitus" OR "Noninsulin-Dependent Diabetes Mellitus" OR "non-insulin-dependent diabetes mellitus" OR "Slow-Onset Diabetes Mellitus" OR "Stable Diabetes Mellitus" OR "T2DM" OR "TIIDM" OR "type 2 (insulin independent) diabetes" OR "Type 2 Diabetes" OR "type 2 diabetes mellitus" OR "type II diabetes" OR "type II diabetes mellitus" OR "unspecified diabetes mellitus") **AND** ("arthritis urica" OR "cheiragra" OR "chiragra" OR "GOUT" OR "Gouts" OR "Arthragra gout" OR "gouty arthritis" OR "gouty arthropathy" OR "gouty attack" OR "hyperuricaemic arthritis" OR "hyperuricemic arthritis" OR "urate inflammation" OR "uric arthritis") **AND** ("Gliflozin" OR "gliflozin derivative" OR "Gliflozins" OR "inhibitor sodium glucose co-transporter 2" OR "inhibitor sodium-glucose transporter 2" OR "inhibitors sodium dependent glucose cotransporter 2" OR "Inhibitors sodium glucose cotransporte" OR "SGLT 2 Inhibitor" OR "SGLT 2 Inhibitors" OR "SGLT2" OR "SGLT-2 Inhibitor" OR "SGLT-2 Inhibitors" OR "sodium glucose cotransporter 2 inhibitor" OR "Sodium Glucose Transporter 2 Inhibitor" OR "Sodium Glucose Transporter 2 Inhibitors" OR " Sodium-Glucose Transporter 2 Inhibitor" OR "SODIUM-GLUCOSE TRANSPORTER 2 INHIBITORS") | | | | | 33 | | | | |
| Scopus | ("adult onset diabetes mellitus" OR "Adult-Onset Diabetes Mellitus" OR "diabetes" OR "DIABETES MELLITUS" OR "diabetes mellitus type 2" OR "diabetes mellitus type ii" OR "DIABETES MELLITUS, TYPE 2" OR "diabetes type 2" OR "diabetes type II" OR "diabetic" OR "diabets" OR "dm 2" OR "insulin independent diabetes" OR "insulin independent diabetes mellitus" OR "ketosis resistant diabetes mellitus" OR "Ketosis-Resistant Diabetes Mellitus" OR "Maturity Onset Diabetes" OR "maturity onset diabetes mellitus" OR "Maturity-Onset Diabetes" OR "Maturity-Onset Diabetes Mellitus" OR "MODY" OR "NIDDM" OR "niddm" OR "NIDDM (non insulin dependent diabetes mellitus)" OR "non insulin dependent (type 2) diabetes mellitus" OR "non insulin dependent diabetes" OR "non insulin dependent diabetes mellitus" OR "non insulin dependent diabetes mellitus" OR "adult onset diabetes" OR "noninsulin dependent (type 2) diabetes mellitus" OR "noninsulin dependent diabetes" OR "Noninsulin Dependent Diabetes Mellitus" OR "Noninsulin-Dependent Diabetes Mellitus" OR "non-insulin-dependent diabetes mellitus" OR "Slow-Onset Diabetes Mellitus" OR "Stable Diabetes Mellitus" OR "T2DM" OR "TIIDM" OR "type 2 (insulin independent) diabetes" OR "Type 2 Diabetes" OR "type 2 diabetes mellitus" OR "type II diabetes" OR "type II diabetes mellitus" OR "unspecified diabetes mellitus") **AND** ("arthritis urica" OR "cheiragra" OR "chiragra" OR "GOUT" OR "Gouts" OR "Arthragra gout" OR "gouty arthritis" OR "gouty arthropathy" OR "gouty attack" OR "hyperuricaemic arthritis" OR "hyperuricemic arthritis" OR "urate inflammation" OR "uric arthritis") **AND** ("Gliflozin" OR "gliflozin derivative" OR "Gliflozins" OR "inhibitor sodium glucose co-transporter 2" OR "inhibitor sodium-glucose transporter 2" OR "inhibitors sodium dependent glucose cotransporter 2" OR "Inhibitors sodium glucose cotransporte" OR "SGLT 2 Inhibitor" OR "SGLT 2 Inhibitors" OR "SGLT2" OR "SGLT-2 Inhibitor" OR "SGLT-2 Inhibitors" OR "sodium glucose cotransporter 2 inhibitor" OR "Sodium Glucose Transporter 2 Inhibitor" OR "Sodium Glucose Transporter 2 Inhibitors" OR " Sodium-Glucose Transporter 2 Inhibitor" OR "SODIUM-GLUCOSE TRANSPORTER 2 INHIBITORS") | | | | | | 113 | | | |
| Web of  Science | ("adult onset diabetes mellitus" OR "Adult-Onset Diabetes Mellitus" OR "diabetes" OR "DIABETES MELLITUS" OR "diabetes mellitus type 2" OR "diabetes mellitus type ii" OR "DIABETES MELLITUS, TYPE 2" OR "diabetes type 2" OR "diabetes type II" OR "diabetic" OR "diabets" OR "dm 2" OR "insulin independent diabetes" OR "insulin independent diabetes mellitus" OR "ketosis resistant diabetes mellitus" OR "Ketosis-Resistant Diabetes Mellitus" OR "Maturity Onset Diabetes" OR "maturity onset diabetes mellitus" OR "Maturity-Onset Diabetes" OR "Maturity-Onset Diabetes Mellitus" OR "MODY" OR "NIDDM" OR "niddm" OR "NIDDM (non insulin dependent diabetes mellitus)" OR "non insulin dependent (type 2) diabetes mellitus" OR "non insulin dependent diabetes" OR "non insulin dependent diabetes mellitus" OR "non insulin dependent diabetes mellitus" OR "adult onset diabetes" OR "noninsulin dependent (type 2) diabetes mellitus" OR "noninsulin dependent diabetes" OR "Noninsulin Dependent Diabetes Mellitus" OR "Noninsulin-Dependent Diabetes Mellitus" OR "non-insulin-dependent diabetes mellitus" OR "Slow-Onset Diabetes Mellitus" OR "Stable Diabetes Mellitus" OR "T2DM" OR "TIIDM" OR "type 2 (insulin independent) diabetes" OR "Type 2 Diabetes" OR "type 2 diabetes mellitus" OR "type II diabetes" OR "type II diabetes mellitus" OR "unspecified diabetes mellitus") **AND** ("arthritis urica" OR "cheiragra" OR "chiragra" OR "GOUT" OR "Gouts" OR "Arthragra gout" OR "gouty arthritis" OR "gouty arthropathy" OR "gouty attack" OR "hyperuricaemic arthritis" OR "hyperuricemic arthritis" OR "urate inflammation" OR "uric arthritis") **AND** ("Gliflozin" OR "gliflozin derivative" OR "Gliflozins" OR "inhibitor sodium glucose co-transporter 2" OR "inhibitor sodium-glucose transporter 2" OR "inhibitors sodium dependent glucose cotransporter 2" OR "Inhibitors sodium glucose cotransporte" OR "SGLT 2 Inhibitor" OR "SGLT 2 Inhibitors" OR "SGLT2" OR "SGLT-2 Inhibitor" OR "SGLT-2 Inhibitors" OR "sodium glucose cotransporter 2 inhibitor" OR "Sodium Glucose Transporter 2 Inhibitor" OR "Sodium Glucose Transporter 2 Inhibitors" OR " Sodium-Glucose Transporter 2 Inhibitor" OR "SODIUM-GLUCOSE TRANSPORTER 2 INHIBITORS") | | | | | | | | 47 | |
| LILACS  BVS | ("adult onset diabetes mellitus" OR "Adult-Onset Diabetes Mellitus" OR "diabetes" OR "DIABETES MELLITUS" OR "diabetes mellitus type 2" OR "diabetes mellitus type ii" OR "DIABETES MELLITUS, TYPE 2" OR "diabetes type 2" OR "diabetes type II" OR "diabetic" OR "diabets" OR "dm 2" OR "insulin independent diabetes" OR "insulin independent diabetes mellitus" OR "ketosis resistant diabetes mellitus" OR "Ketosis-Resistant Diabetes Mellitus" OR "Maturity Onset Diabetes" OR "maturity onset diabetes mellitus" OR "Maturity-Onset Diabetes" OR "Maturity-Onset Diabetes Mellitus" OR "MODY" OR "NIDDM" OR "niddm" OR "NIDDM (non insulin dependent diabetes mellitus)" OR "non insulin dependent (type 2) diabetes mellitus" OR "non insulin dependent diabetes" OR "non insulin dependent diabetes mellitus" OR "non insulin dependent diabetes mellitus" OR "adult onset diabetes" OR "noninsulin dependent (type 2) diabetes mellitus" OR "noninsulin dependent diabetes" OR "Noninsulin Dependent Diabetes Mellitus" OR "Noninsulin-Dependent Diabetes Mellitus" OR "non-insulin-dependent diabetes mellitus" OR "Slow-Onset Diabetes Mellitus" OR "Stable Diabetes Mellitus" OR "T2DM" OR "TIIDM" OR "type 2 (insulin independent) diabetes" OR "Type 2 Diabetes" OR "type 2 diabetes mellitus" OR "type II diabetes" OR "type II diabetes mellitus" OR "unspecified diabetes mellitus" OR "Diabete" OR "Diabete Melito" OR "Diabetes" OR "Diabetes do Tipo 2" OR "Diabetes Melito" OR "Diabetes Mellitus" OR "Diabetes Mellitus de Inicio Adulto" OR "Diabetes Mellitus de Inicio en la Madurez" OR "Diabetes Mellitus de Início Gradativo" OR "Diabetes Mellitus de Inicio Lento" OR "Diabetes Mellitus de Início na Maturidade" OR "Diabetes Mellitus de Início no Adulto" OR "Diabetes Mellitus Estable" OR "Diabetes Mellitus Estável" OR "Diabetes Mellitus não Dependente de Insulina" OR "Diabetes Mellitus não Insulinodependente" OR "Diabetes Mellitus não Insulino-Dependente" OR "Diabetes Mellitus no Insulinodependiente" OR "Diabetes Mellitus no Insulino-Dependiente" OR "Diabetes Mellitus Resistente a Cetose" OR "Diabetes Mellitus Resistente a la Cetosis" OR "Diabetes Mellitus Tipo 2" OR "DMNID" OR "Diabetes Mellitus Tipo II" OR "Diabetes Tipo 2" OR "DMNID" OR "MODY" OR "Diabetes Mellitus Tipo 2" OR "DMIM") AND ("arthritis urica" OR "cheiragra" OR "chiragra" OR "GOUT" OR “Gota” OR "Gouts" OR "Arthragra gout" OR "gouty arthritis" OR "gouty arthropathy" OR "gouty attack" OR "hyperuricaemic arthritis" OR "hyperuricemic arthritis" OR "urate inflammation" OR "uric arthritis") AND ("Gliflozin" OR "Gliflozina" OR "Gliflozinas" OR "Gliflozins" OR "Inhibidor de SGLT2" OR "Inhibidor de SGLT-2" OR "Inhibidor del Transportador 2 de Glucosa y Sodio" OR "Inhibidores de SGLT 2" OR "Inhibidores de SGLT-2" OR "Inhibidores del Cotransportador de Sodio-Glucosa 2" OR "Inhibidores del Cotransportador de Sodio-Glucosa de Tipo 2" OR "Inhibidores del SGLT 2" OR "Inhibidores del SGLT-2" OR "Inhibidores del Transportador de Sodio-Glucosa 2" OR "Inhibidores del Transportador de Sodio-Glucosa de Tipo 2" OR "Inhibidores SGLT 2" OR "Inhibidores SGLT2" OR "Inhibidores SGLT-2" OR "Inhibitor Sodium Glucose Transporter 2" OR "Inhibitors Sodium-Glucose Transporter 2" OR "Inibidor do SGLT2" OR "Inibidor do SGLT-2" OR "Inibidor do Transportador 2 de Sódio-Glicose" OR "Inibidor do Transportador de Sódio-Glicose 2" OR "Inibidores do SGLT2" OR "Inibidores do SGLT-2" OR "SGLT 2 Inhibitor" OR "SGLT 2 Inhibitors" OR "SGLT2 Inhibitor" OR "SGLT-2 Inhibitor" OR "SGLT-2 Inhibitors" OR "SGLT2 Inhibitors Sodium Glucose Transporter 2" OR "Sodium-Glucose Transporter 2 Inhibitors") | | | | | | | 42 | | |
| BDTD | ("adult onset diabetes mellitus" OR "Adult-Onset Diabetes Mellitus" OR "diabetes" OR "DIABETES MELLITUS" OR "diabetes mellitus type 2" OR "diabetes mellitus type ii" OR "DIABETES MELLITUS, TYPE 2" OR "diabetes type 2" OR "diabetes type II" OR "diabetic" OR "diabets" OR "dm 2" OR "insulin independent diabetes" OR "insulin independent diabetes mellitus" OR "ketosis resistant diabetes mellitus" OR "Ketosis-Resistant Diabetes Mellitus" OR "Maturity Onset Diabetes" OR "maturity onset diabetes mellitus" OR "Maturity-Onset Diabetes" OR "Maturity-Onset Diabetes Mellitus" OR "MODY" OR "NIDDM" OR "niddm" OR "NIDDM (non insulin dependent diabetes mellitus)" OR "non insulin dependent (type 2) diabetes mellitus" OR "non insulin dependent diabetes" OR "non insulin dependent diabetes mellitus" OR "non insulin dependent diabetes mellitus" OR "adult onset diabetes" OR "noninsulin dependent (type 2) diabetes mellitus" OR "noninsulin dependent diabetes" OR "Noninsulin Dependent Diabetes Mellitus" OR "Noninsulin-Dependent Diabetes Mellitus" OR "non-insulin-dependent diabetes mellitus" OR "Slow-Onset Diabetes Mellitus" OR "Stable Diabetes Mellitus" OR "T2DM" OR "TIIDM" OR "type 2 (insulin independent) diabetes" OR "Type 2 Diabetes" OR "type 2 diabetes mellitus" OR "type II diabetes" OR "type II diabetes mellitus" OR "unspecified diabetes mellitus" OR "Diabete" OR "Diabete Melito" OR "Diabetes" OR "Diabetes do Tipo 2" OR "Diabetes Melito" OR "Diabetes Mellitus" OR "Diabetes Mellitus de Inicio Adulto" OR "Diabetes Mellitus de Inicio en la Madurez" OR "Diabetes Mellitus de Início Gradativo" OR "Diabetes Mellitus de Inicio Lento" OR "Diabetes Mellitus de Início na Maturidade" OR "Diabetes Mellitus de Início no Adulto" OR "Diabetes Mellitus Estable" OR "Diabetes Mellitus Estável" OR "Diabetes Mellitus não Dependente de Insulina" OR "Diabetes Mellitus não Insulinodependente" OR "Diabetes Mellitus não Insulino-Dependente" OR "Diabetes Mellitus no Insulinodependiente" OR "Diabetes Mellitus no Insulino-Dependiente" OR "Diabetes Mellitus Resistente a Cetose" OR "Diabetes Mellitus Resistente a la Cetosis" OR "Diabetes Mellitus Tipo 2" OR "DMNID" OR "Diabetes Mellitus Tipo II" OR "Diabetes Tipo 2" OR "DMNID" OR "MODY" OR "Diabetes Mellitus Tipo 2" OR "DMIM") AND ("arthritis urica" OR "cheiragra" OR "chiragra" OR "GOUT" OR “Gota” OR "Gouts" OR "Arthragra gout" OR "gouty arthritis" OR "gouty arthropathy" OR "gouty attack" OR "hyperuricaemic arthritis" OR "hyperuricemic arthritis" OR "urate inflammation" OR "uric arthritis") AND ("Gliflozin" OR "Gliflozina" OR "Gliflozinas" OR "Gliflozins" OR "Inhibidor de SGLT2" OR "Inhibidor de SGLT-2" OR "Inhibidor del Transportador 2 de Glucosa y Sodio" OR "Inhibidores de SGLT 2" OR "Inhibidores de SGLT-2" OR "Inhibidores del Cotransportador de Sodio-Glucosa 2" OR "Inhibidores del Cotransportador de Sodio-Glucosa de Tipo 2" OR "Inhibidores del SGLT 2" OR "Inhibidores del SGLT-2" OR "Inhibidores del Transportador de Sodio-Glucosa 2" OR "Inhibidores del Transportador de Sodio-Glucosa de Tipo 2" OR "Inhibidores SGLT 2" OR "Inhibidores SGLT2" OR "Inhibidores SGLT-2" OR "Inhibitor Sodium Glucose Transporter 2" OR "Inhibitors Sodium-Glucose Transporter 2" OR "Inibidor do SGLT2" OR "Inibidor do SGLT-2" OR "Inibidor do Transportador 2 de Sódio-Glicose" OR "Inibidor do Transportador de Sódio-Glicose 2" OR "Inibidores do SGLT2" OR "Inibidores do SGLT-2" OR "SGLT 2 Inhibitor" OR "SGLT 2 Inhibitors" OR "SGLT2 Inhibitor" OR "SGLT-2 Inhibitor" OR "SGLT-2 Inhibitors" OR "SGLT2 Inhibitors Sodium Glucose Transporter 2" OR "Sodium-Glucose Transporter 2 Inhibitors") | | | | | | | | | 0 |
| ProQuest | ("adult onset diabetes mellitus" OR "Adult-Onset Diabetes Mellitus" OR "diabetes" OR "DIABETES MELLITUS" OR "diabetes mellitus type 2" OR "diabetes mellitus type ii" OR "DIABETES MELLITUS, TYPE 2" OR "diabetes type 2" OR "diabetes type II" OR "diabetic" OR "diabets" OR "dm 2" OR "insulin independent diabetes" OR "insulin independent diabetes mellitus" OR "ketosis resistant diabetes mellitus" OR "Ketosis-Resistant Diabetes Mellitus" OR "Maturity Onset Diabetes" OR "maturity onset diabetes mellitus" OR "Maturity-Onset Diabetes" OR "Maturity-Onset Diabetes Mellitus" OR "MODY" OR "NIDDM" OR "niddm" OR "NIDDM (non insulin dependent diabetes mellitus)" OR "non insulin dependent (type 2) diabetes mellitus" OR "non insulin dependent diabetes" OR "non insulin dependent diabetes mellitus" OR "non insulin dependent diabetes mellitus" OR "adult onset diabetes" OR "noninsulin dependent (type 2) diabetes mellitus" OR "noninsulin dependent diabetes" OR "Noninsulin Dependent Diabetes Mellitus" OR "Noninsulin-Dependent Diabetes Mellitus" OR "non-insulin-dependent diabetes mellitus" OR "Slow-Onset Diabetes Mellitus" OR "Stable Diabetes Mellitus" OR "T2DM" OR "TIIDM" OR "type 2 (insulin independent) diabetes" OR "Type 2 Diabetes" OR "type 2 diabetes mellitus" OR "type II diabetes" OR "type II diabetes mellitus" OR "unspecified diabetes mellitus") **AND** ("arthritis urica" OR "cheiragra" OR "chiragra" OR "GOUT" OR "Gouts" OR "Arthragra gout" OR "gouty arthritis" OR "gouty arthropathy" OR "gouty attack" OR "hyperuricaemic arthritis" OR "hyperuricemic arthritis" OR "urate inflammation" OR "uric arthritis") **AND** ("Gliflozin" OR "gliflozin derivative" OR "Gliflozins" OR "inhibitor sodium glucose co-transporter 2" OR "inhibitor sodium-glucose transporter 2" OR "inhibitors sodium dependent glucose cotransporter 2" OR "Inhibitors sodium glucose cotransporte" OR "SGLT 2 Inhibitor" OR "SGLT 2 Inhibitors" OR "SGLT2" OR "SGLT-2 Inhibitor" OR "SGLT-2 Inhibitors" OR "sodium glucose cotransporter 2 inhibitor" OR "Sodium Glucose Transporter 2 Inhibitor" OR "Sodium Glucose Transporter 2 Inhibitors" OR " Sodium-Glucose Transporter 2 Inhibitor" OR "SODIUM-GLUCOSE TRANSPORTER 2 INHIBITORS") | | | | | | | | | 0 |
