# Supplementary material for: Effects of SGLT2 inhibitors on gout-related and cardiometabolic outcomes in patients with type 2 diabetes mellitus: a scoping review
Source: Adv Rheumatol. 2026 Jul 24;66:56. doi: 10.1186/s42358-026-00564-w (PMC13632354; doi:10.1186/s42358-026-00564-w)
Supplement: Supplementary file 3 [file 42358_2026_564_MOESM3_ESM.docx]

**Effects of SGLT2 inhibitors on gout-related and cardiometabolic outcomes in patients with type 2 diabetes mellitus: a scoping review.**

**Additional file 3**

Study characteristics: authors, country, journal, design, population, sample, and follow-up

| **Study** | **Author and year** | **Country** | **Journal** | **Study design** | **Population and study sample** | **Follow- up (years)** |
| --- | --- | --- | --- | --- | --- | --- |
| S1 | Ouchi et al., 2018 [51] | Japan | Diabetes, Obesity, and Metabolism | Integrated analysis | 1,046 with T2DM | 2 |
| S2 | Li et al., 2019 [34] | China | The Lancet.  Rheumatology | Post hoc analysis (CANVAS  program) | 10,142 with T2DM | 3,6 |
| S3 | Fralick et al., 2020 [42] | USA | Annals of Internal Medicine | Retrospective cohort study | 205,907 with T2DM | 0,8 |
| S4 | Chung et al., 2021 [41] | Taiwan | JAMA Network Open. | Retrospective cohort study | 231,208 with T2DM | 2,5 |
| S5 | Lund et al., 2021 [43] | Denmark | Pharmacoepidemiology and Drug Safety | Retrospective cohort study with PSM and SA | 22,094 with T2DM | 3 |
| S6 | Doehner et al., 2022 [40] | Germany | European Heart Journal | Post hoc analysis (EMPEROR-  Reduced trial) | 3,676 with HFrEF with or without T2DM | 1,3 |
| S7 | Ferreira et al., 2022 [33] | France | Diabetes, Obesity, and Metabolism | Post hoc analysis (EMPA-REG  OUTCOME trial) | 7,020 with T2DM | 2,6 |
| S8 | Gouda et al., 2022 [52] | Japan | Journal of Diabetes Investigation. | Cross-sectional study with PSM | 856,796 with T2DM  without prior use of antihypertensive, anti- gout or anti- | 2 |
|  |  |  |  |  | dyslipidemic drugs for  ≥1 year |  |
| S9 | McDowell et al., 2022 [48] | United Kingdom | European Journal of Heart Failure. | Post hoc analysis (DAPA-HF) | 3,119 with HFrEF with or without T2DM | 1,1 |
| S10 | Butt et al., 2023 [49] | United Kingdom | JAMA Cardiology | Post hoc analysis (DAPA-HF and DELIVER) | 11,005 with HF with or without T2DM | 1,8 |
| S11 | McCormick et al., 2023 [37] | Canada | Annals of Internal Medicine | Retrospective cohort study | 8,150 with gout and T2DM | 1,6 |
| S12 | Subramanian et al., 2023  [39] | United Kingdom | Diabetes, Obesity, and Metabolism | Retrospective cohort study | 43,043 with T2DM | 1,7 |
| S13 | Wei et al., 2023 [38] | United Kingdom | JAMA Network Open | Retrospective cohort study | 5,931 with gout and T2DM | 2,7 |
| S14 | Wood et al., 2023 [44] | USA | Clinical Rheumatology | Retrospective cohort study with SSA | 27,158 with T2DM | 2 |
| S15 | Zhou et al., 2023 [23] | China | Rheumatology | Retrospective cohort study | 43,201 with T2DM | 5,6 |
| S16 | Doehner et al., 2024 [50] | Germany | JACC Heart Fail | Post hoc analysis (EMPEROR-  Preserved trial) | 5,924 with HFpEF | 2 |
| S17 | McCormick et al., 2024 [45] | Canada | BMJ: British Medical Journal / British Medical Association. | Retrospective cohort study | 20,144 with T2DM and nephrolithiasis | 1,2 |
| S18 | McCormick et al., 2024 [35] | Canada | JAMA Internal Medicine. | Retrospective cohort study | 34,604 with T2DM  using metformin | 1,4 |
| S19 | Preston et al., 2024 [46] | United Kingdom | Clinical Therapeutics | Retrospective cohort study with PSM | 2,890,000 with T2DM | 5 |
| S20 | Sridhar et al., 2024 [31] | Canada | Diabetes, Obesity, and Metabolism | Post hoc analysis (VERTIS CV Trial) | 8,246 with T2DM | 3,6 |
| S21 | Yokose et al., 2024 [32] | USA | Seminars in Arthritis and Rheumatism | Retrospective cohort study | 56 with gout and with or without T2DM | 6 |
| S22 | Yokoyama et al., 2024 [47] | Japan | Biological & Pharmaceutical  Bulletin | Self-controlled SSA | 12,396 SGLT2i  initiators and uric acid- lowering agents | 2 |

**Abbreviations:** PSM - Propensity score matching; SA - Symmetry analysis; SSA - Sequence symmetry analysis; T2DM - Type 2 diabetes mellitus; SGLT2i - Sodium-glucose cotransporter 2 inhibitor; GLP1-RA

- Glucagon like peptide-1 receptor agonists; DPP4i - Dipeptidyl peptidase-4 inhibitors; HF - Heart failure; HfrEF - HF with reduced ejection fraction; HFpEF - Heart failure with preserved ejection fraction USA - United States of America; CANVAS - Canagliflozin Assessment in Nephropathy and Vascular Adiseases Study; EMPEROR - Empagliflozin outcome trial in patients with chronic heart failure; DAPA- HF - Dapagliflozin and the prevention of adverse outcomes in heart failure; DELIVER - Dapagliflozin evaluation to improve the lives of individuals with ventricular dysfunction; VERTIS CV - Cardiovascular outcomes following ertugliflozin treatment in type 2 diabetes mellitus participants with vascular disease
