# Supplementary material for: Effects of SGLT2 inhibitors on gout-related and cardiometabolic outcomes in patients with type 2 diabetes mellitus: a scoping review
Source: Adv Rheumatol. 2026 Jul 24;66:56. doi: 10.1186/s42358-026-00564-w (PMC13632354; doi:10.1186/s42358-026-00564-w)
Supplement: Supplementary file 4 [file 42358_2026_564_MOESM4_ESM.docx]

**Effects of SGLT2 inhibitors on gout-related and cardiometabolic outcomes in patients with type 2 diabetes mellitus: a scoping review.**

**Additional file 4**

Baseline characteristics: age, sex, ethnicity, gout, CV comorbidities, and CKD.

| **Study** | **Author and year** | **Mean age (with SD)** | **Female (%)** | **Ethnicity** | **% gout at baseline** | **CV comorbidities and CKD (%)** |
| --- | --- | --- | --- | --- | --- | --- |
| S1 | Ouchi et al., 2018 [51] | 58.3 ± 10.3 | 30 | Asians | NR | NR |
| S2 | Li et al., 2019 [34] | 63 ± 8 | 36 | White (78%) | 5 | HTN 9; CAD 56; HF 15; CVD 19 |
| S3 | Fralick et al., 2020 [42] | 54.62 ± 9.73 | 52 | NR | Zero | HTN 70; DLP 68; CAD 9,3; CVD 1,2;  HF 2,2; Overweight or obesity 30 |
| S4 | Chung et al., 2021 [41] | 61.53 ±  12,86 | 49 | Asians | Zero | HTN 10; DLP 66.97; CVD 1.8; CAD  16,69; Obesity 1,46; CKD 11,53 |
| S5 | Lund et al., 2021 [43] | 61 ± 8 | 43 | White | NR | HTN 37; Obesity 25; CKD 2; HA 8;  HF 5 |
| S6 | Doehner et al., 2022 [40] | 66.7 ± 11.6 | 24 | White (70%) | NR;  53% with hyperuricemia | HTN 70; T2DM 50 |
| S7 | Ferreira et al., 2022 [33] | 63,0 ± 8,7 | 30 | White (72%) | 5.9 | HTN 71; HF 10 |
| S8 | Gouda et al., 2022 [52] | 51.4 ± 7.4 | 18 | Asians | NR | HA 1; HF 5; CVD 8; Obesity 20;  CKD1 |
| S9 | McDowell et al., 2022 [48] | 67.3 ± 10.4 | 20 | White (80%) | 10.3 | CKD 40; T2DM 40 |
| S10 | Butt et al., 2023 [49] | 69.3 ± 10.6 | 37%  (without gout); 20%  (with gout) | White (70%) | 10.1 | HTN 85; T2DM 45; HA 30; CVD 10;  Obesity 45; CKD 50 |
| S11 | McCormick et al., 2023 [37] | 64.11 ±  11.26 | 30 | NR | 100 | HTN 90; Obesity 13; HA 17; CVD 19;  HF 23; CKD 25 |
| S12 | Subramanian et al., 2023 [39] | 57.71 ±  10.64 | 43 | White (50%)  NR (45%) | Zero | Obesity 70; HTN 55; CAD13; HA 6;  CVD 3; CKD 5 (stage ≥ 4) |
| S13 | Wei et al., 2023 [38] | 66.0 ± 11.6 | 18 | White | 100 | HTN 70; HA 9; CVD 4; HF 8;  CAD 20; CKD 11 |
| S14 | Wood et al., 2023 [44] | 65.4 ± 10 | 4 | White (74%) | NR | NR |
| S15 | Zhou et al., 2023 [23] | 63,23 ± 8.02 | 45 | Asians | Zero | HTN 26; HF 2,5; DLP 4; CAD 14;  HA 3,5; CVD 3; Obesity 2; CKD 5 |
| S16 | Doehner et al., 2024 [50] | 72 ± 9.8 | 45 | White (75%) | 5% with gout;  15% using anti-gout medications; 49% with hyperuricemia | HTN 90; T2DM 50; Obesity 45; |
| S17 | McCormick et al., 2024 [45] | 62.53 ±  11.45 | 45 | NR | 22 | HTN 65, Obesity 15; CAD 43; HA 15;  CVD 13; HF 15; CKD 17 |
| S18 | McCormick et al., 2024 [35] | 60 ± 12.4 | 40 | NR | 15.6 | HTN 66; Obesity 10; HA 10; CVD 9;  HF 10; CAD 40; CKD 8 |
| S19 | Preston et al., 2024 [46] | 62.7 ± 12.1 | 40 | NR | NR | HTN 80; Obesity 30; DLP 60; HF 20;  CAD 40; CKD 20 |
| S20 | Sridhar et al., 2024 [31] | 64.6 ± 8.2 | 30 | White (90%) | 7.5 | HTN 95; DLP 90 |
| S21 | Yokose et al.,  2024 [32] | 66.8 ± 9.8 | 10 | NR | 100 | CKD 18; HF 18 |
| S22 | Yokoyama et al., 2024 [47] | 53 ± 6 | 25 | Asians | NR | NR |

**Abbreviations:** CV - Cardiovascular; CKD - Chronic kidney disease; NR - Not reported; HTN - Hypertension; CAD - Coronary artery disease; CVD - Cerebrovascular disease; HF - Heart failure; DLP - Dyslipidemia; HA - Heart attack; SD - Standard deviation
